# Supplementary material for: A Unified Software/Hardware Scalable Architecture for Brain-Inspired Computing Based on Self-Organizing Neural Models
Source: Front Neurosci. 2022 Mar 2;16:825879. doi: 10.3389/fnins.2022.825879 (PMC8926299; doi:10.3389/fnins.2022.825879)
Supplement: Supplementary file 1 [file Data_Sheet_1.PDF]

Supplementary Material

1 SUPPLEMENTARY DATA

2 SUPPLEMENTARY TABLES AND FIGURES

2.1 Figures

2.2 Tables

Table S1. The best model hyperparameters. The table is references in by the subsection 5.1.3.

Hyperparameter	MNIST SOM (10x10)	S-MNIST SOM (16x16)	F-MNIST SOM (10x10)	Gests SOM (16x16)
Unimodal parameteres				
σ_i	0.532	0.236	0.777	1.461
σ_f	0.003	0.005	0.004	0.048
ϵ_i	1.554	3.203	0.944	13.523
ϵ_f	0.002	0.092	0.002	0.057
α	0.245	0.06	0.264	0.081
Reentry parameteres				
μ	1.	1.	1.	1.
α_{train}	30.414	19.796	71.576	26.414
α_{test}	2.605	5.477	0.727	1.256
only BMU may be prediction	True	True	True	True
